# sMEK1 inhibits endothelial cell proliferation by attenuating VEGFR-2-dependent-Akt/eNOS/HIF-1α signaling pathways

**DOI:** 10.18632/oncotarget.5570

**Published:** 2015-09-10

**Authors:** Boh-Ram Kim, Seung Hee Seo, Mi Sun Park, Seung-Hoon Lee, Youngjoo Kwon, Seung Bae Rho

**Affiliations:** ^1^ Research Institute, National Cancer Center, Ilsan-ro, Ilsandong-gu, Goyang-si Gyeonggi-do, Republic of Korea; ^2^ Department of Life Science, Yong In University, Samga-dong, Cheoin-gu, Yongin-si Gyeonggi-do, Republic of Korea; ^3^ College of Pharmacy, Graduate School of Pharmaceutical Sciences, Ewha Global Top 5 Program, Ewha Womans University, Seoul, Republic of Korea

**Keywords:** sMEK1 tumor suppressor, hypoxic condition, anti-angiogenic activity, ovarian tumor, xenograft model

## Abstract

The suppressor of MEK null (sMEK1) protein possesses pro-apoptotic activities. In the current study, we reveal that sMEK1 functions as a novel anti-angiogenic factor by suppressing vascular endothelial growth factor (VEGF)-induced cell proliferation, migration, and capillary-like tubular structure *in vitro*. In addition, sMEK1 inhibited the phosphorylation of the signaling components up- and downstream of Akt, including phospholipase Cγ1 (PLC-γ1), 3-phosphoinositide-dependent protein kinase 1 (PDK1), endothelial nitric oxide synthetase (eNOS), and hypoxia-inducible factor 1 (HIF-1α) during ovarian tumor progression via binding with vascular endothelial growth factor receptor 2 (VEGFR-2). Furthermore, sMEK1 decreased tumor vascularity and inhibited tumor growth in a xenograft human ovarian tumor model. These results supply convincing evidence that sMEK1 controls endothelial cell function and subsequent angiogenesis by suppressing VEGFR-2-mediated PI3K/Akt/eNOS signaling pathway. Taken together, our results clearly suggest that sMEK1 might be a novel anti-angiogenic and anti-tumor agent for use in ovarian tumor.

## INTRODUCTION

Angiogenesis is complex and involves multiple processes, such as tumor growth and metastasis, that are regulated by various pro- and anti-angiogenic factors, including angiogenic proteins, apoptotic regulators, and hormone metabolites. Cancer cells secrete various growth factors, including vascular endothelial growth factor (VEGF), basic fibroblast growth factor (bFGF), and platelet-derived growth factor (PDGF), which activate endothelial cells to stimulate the formation of new blood vessels [1–8]. VEGF is the most important pro-angiogenic factor, and its signaling cascade is one of the major pathways activating this process [9, 10]. The VEGF family consists of seven members (VEGF-A through F and PIGF), which each contains a common VEGF homology domain, such as the 45-kDa heparin-binding homodimeric glycoprotein domain. VEGF-A binds directly to and activates three receptor tyrosine kinases (RTK): VEGFR-1 (Flt-1), -2 (KDR/Flk-1), and -3 (Flt-4). These receptors control pathological as well as physiological angiogenesis [11, 12]. After binding to VEGF receptors on the surface of endothelial cells, VEGF activates signaling pathways, including PI3K/Akt/mTOR, which subsequently activates endothelial cell recruitment and proliferation [13–16]. Interestingly, VEGFR-1 is the major receptor involved in developmental angiogenesis, but it does not appear to be essential for pathogenic angiogenesis [14]. In contrast, VEGFR-2 has potent tyrosine kinase activity and functions as the major signal transducer in tumor angiogenesis, which involves vascular cell proliferation, migration, and invasion [17–19]. Shalaby et al. [20] reported that VEGFR-2-deficient mice were embryonic lethal because their endothelial cells did not form a structured, organized blood vessel network. In addition, Flk-1-deficient mice revealed that Flk-1 is essential for the development of hematopoietic stem cells in embryos, but not for the function of hematopoietic stem cells in adult mouse bone marrow [21].

Hypoxia plays a major role in tumor development, progression of vascular-associated diseases, and subsequent pathological angiogenesis involving the upregulation of VEGF and VEGF-associated molecules [18, 22, 23]. Hypoxia-inducible factor-1α (HIF-1α) is a well validated target protein found in various malignant tumors. It is a major regulator of several molecules important for tumor angiogenesis, including those that mediate embryonic development, cell metabolism, tumor growth, apoptosis, and metastasis [24–30]. HIF-1α is upregulated in various types of human malignant tumors including brain, cervical, pancreatic, liver, gastric, bladder, breast, and ovarian cancers [31–35]. In addition, its cellular activity influences both tumor angiogenesis and tumorigenesis [36–38].

The sMEK1 tumor suppressor protein, also known as protein phosphatase 4 regulatory subunit 3 (PP4R3), is a major regulator of cellular functions including apoptosis, cell growth, microtubule organization, cell cycle arrest, and TNF and PI3K/Akt signaling [39–43]. To exert these effects, sMEK1 binds to various intracellular proteins, such as ATP-dependent chaperonin [44], histone deacetylase 3 (HDAC3) [41, 45], insulin receptor substrate 4 (IRS-4) [46], and target of rapamycin (TOR) [47]. Previous studies demonstrated that the overexpression of sMEK1 increases hepatic gluconeogenesis, whereas knockdown of sMEK1 decreases blood glucose levels and increases hepatic CRTC2 phosphorylation. In accordance, a sMEK1 suppressor was found to be an essential regulator of hepatic gluconeogenesis [48]. Recently, Dong et al. [49] reported that sMEK1 increases the pro-apoptotic activity of the tumor suppressor BLU via physical interaction. In addition, the expression of sMEK1 was decreased in ovarian and cervical tumor tissues and tumor cell lines, and the sMEK1 gene was hypermethylated.

In this study, we demonstrated the anti-angiogenic effects of sMEK1 using *in vitro* human umbilical vein endothelial cells (HUVECs) and SKOV-3 ovarian cancer cells, and *in vivo* mouse model systems. However, the mechanisms underlying sMEK1-induced angiogenesis remain unclear. Given our observations indicating a role for sMEK1 in tumor angiogenesis, we sought to determine the direct effects of sMEK1 biological function in ovarian tumorigenesis.

## RESULTS

### A physical interaction between sMEK1 and VEGFR-2, but not VEGFR-1

**Figure 1:**
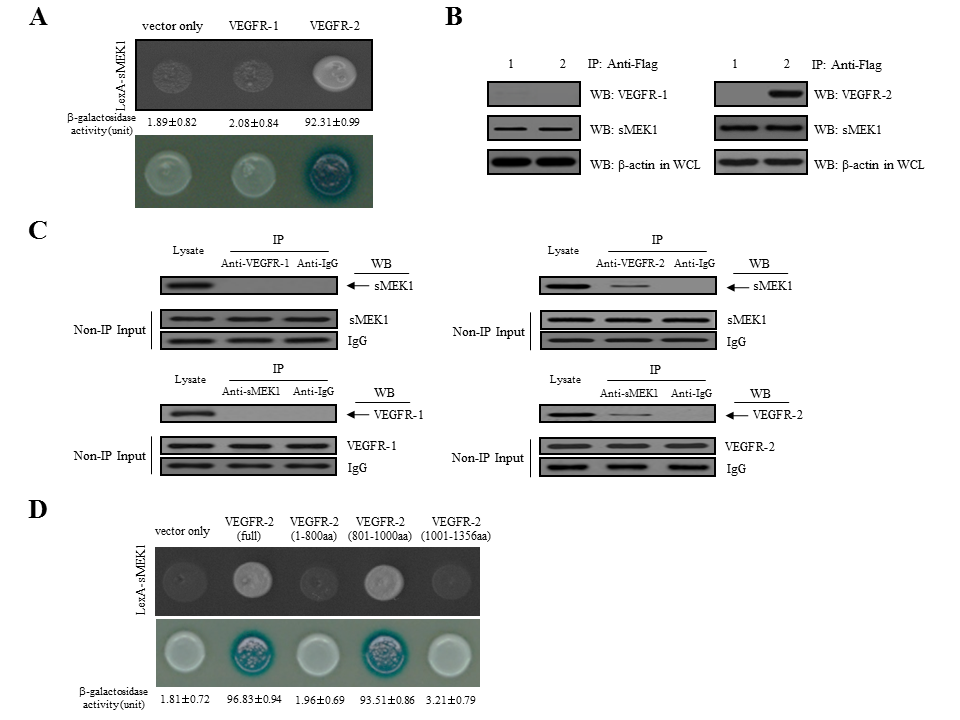
Physical interaction between sMEK1 and VEGFR-2 **A.** Positive interactions were confirmed by observed cell growth on a medium lacking leucine and by the formation of blue colonies on X-gal plates containing 2% galactose. β-galactosidase activity (unit), measured by adding *o*-nitrophenyl β-_D_-galactopyranoside (ONPG), is shown below the corresponding lanes. **B.** The co-immunoprecipitation of sMEK1 with VEGFR-1 or VEGFR-2. Immunoprecipitation from transfected HEK293T cells was performed using anti-FLAG antibodies in lysates, followed by immunoblotting with anti-sMEK1, anti-VEGFR-1, and anti-VEGFR-2 antibodies. **C.** Endogenous proteins in total lysates of HEK293T cells were subjected to co-immunoprecipitation (IP) with an antibody as indicated followed by Western blotting (WB) with an anti-sMEK1 or anti-VEGFR-2 antibody. A rabbit IgG and VEGFR-1 were included as an IP negative control. The input (Non-IP) WB data indicated the integrity of lysates used for IP. **D.** Mapping of the VEGFR-2 region critical for the interaction with sMEK1 using a protein-protein interaction assay *in vivo*. The cDNA constructs were co-transformed into EGY48 yeast cells, and protein-protein interactions were assessed using a yeast two-hybrid system. Positive interactions were confirmed by observed cell growth (upper panel) and the formation of blue colonies (lower panel). The β-galactosidase activity of each construct in negative controls (vector only) was < 1.81±0.72. Data are representative of three independent experiments and are presented as means±SDs.

To assess the biological function of sMEK1 in angiogenesis, we used a yeast two-hybrid system and co-immunoprecipitation assays. We first assessed the intracellular binding of sMEK1 to VEGFR-1 and VEGFR-2. Positive interactions were verified by assessing both cell growth on leucine-deficient plates and β-galactosidase activity using ortho-nitrophenyl-β-galactoside (ONPG). As shown in Figure 1A, β-galactosidase was fully activated (92.31±0.99) in the interaction between sMEK1 and VEGFR-2, but not between sMEK1 and empty vector (vector only; 1.89±0.82) or VEGFR-1 (2.08±0.84). Therefore, VEGFR-1 was used as a negative control in subsequent experiments. We next used co-immunoprecipitation to confirm the direct interaction between sMEK1 and VEGFR-2. DNA constructs expressing sMEK1 (pcDNA3.1/FLAG-sMEK1) and VEGFR-1 or VEGFR-2 (pcDNA3.1-VEGFR-1 or VEGFR-2) or pcDNA3.1/FLAG-sMEK1 and vector only (pcDNA3.1) were co-transfected into HEK293T cells. Immunoprecipitation was then performed in lysates from transfected cells using an anti-FLAG antibody, and the precipitated proteins were immunoblotted using anti-sMEK1, anti-VEGFR-1, or anti-VEGFR-2 antibodies. As seen in Figure 1B, pcDNA3.1-VEGF-2 co-immunoprecipitated with pcDNA3.1/Flag-sMEK1 (lane 2 in the upper right panel), but not with pcDNA3.1 (vector only) or VEGFR-1 (lane 1 in the upper left panel). We then investigated the interaction between endogenous sMEK1 and VEGFR-2. The tumor suppressor sMEK1 binds with VEGFR-2 (right panel), but not VEGFR-1 (left panel) (Figure 1C).

Next, constructs containing three VEGFR-2 deletion fragments were designed to determine the location of the sMEK1-binding region within VEGFR-2 using a yeast two-hybrid assay system. Full-length human sMEK1 and either full-length human VEGFR-2 or one of three truncated mutants (Met^1^-Gly^800^, Leu^801^-Leu^1000^, and Thr^1001^-Val^1356^) were introduced into EGY48 yeast cells. A β-galactosidase assay indicated that the VEGFR-2 region responsible for binding sMEK1 was within amino acids Leu^801^-Leu^1000^ (Figure 1D). Taken together, these results strongly suggest that sMEK1 binds directly with VEGFR-2 under physiological conditions.

### sMEK1 decreases VEGF-stimulated VEGFR-2 phosphorylation (Tyr-951)

**Figure 2:**
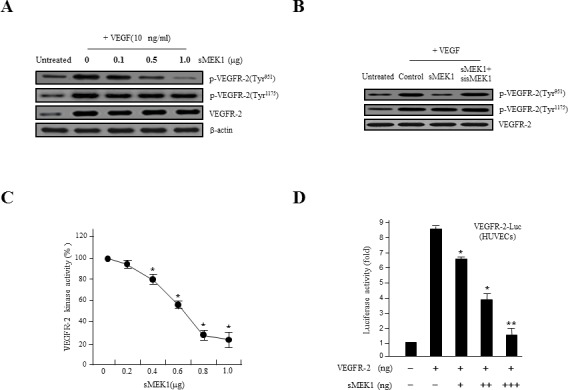

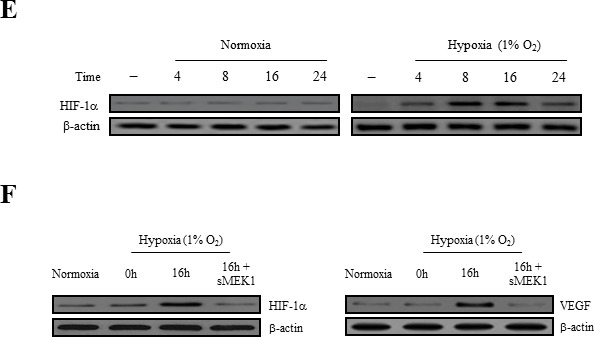
sMEK1 decreases VEGF-stimulated VEGFR-2 phosphorylation (Tyr-951) **A.** HUVECs were transfected with different concentrations of sMEK1, and total cell lysates were prepared. The expression of total and phosphorylated (Tyr-951 or Tyr-1175) VEGFR-2 was measured by immunoblot analysis in cells treated with VEGF. **B.** HUVECs were treated with VEGF and then transfected with the control vector, sMEK1, or sMEK1 plus sisMEK1. sMEK1 suppressed VEGF-stimulated VEGFR-2 (Tyr-951) phosphorylation. VEGFR-2 (Tyr-951 or Tyr-1175) phosphorylation was evaluated using specific antibodies, and VEGFR-2 was used as a loading control. **C.** sMEK1 suppressed VEGFR-2 kinase activity *in vitro*, analyzed using an *in vitro* HTScan VEGFR-2 kinase assay kit followed by colorimetric detection according to the manufacturer's protocols. Values are presented as means±SDs; *, *P* < 0.05 *vs*. control. **D.** sMEK1 suppresses VEGFR-2-dependent transcription. HUVECs were co-transfected with 500 ng VEGFR-2-Luc, 500 ng VEGFR-2 expression plasmid (pcDNA3.1/VEGFR-2), and increasing concentrations of a plasmid encoding sMEK1 (pcDNA3.1/Flag-sMEK1) (50, 250, and 500 ng). Each data point represents triplicate samples, and the bars indicate means±SDs. *, *P* < 0.05; **, *P* < 0.01 *vs*. control. The experiments were repeated three times with similar results. **E.** HUVECs were seeded and then grown under normoxic or hypoxic conditions for 4, 8, 16, or 24 h. HIF-1α protein accumulation was measured by immunoblot analysis. **F.** sMEK1 inhibits hypoxia-induced VEGF and HIF-1α activation. HUVECs were transfected with sMEK1 for 16 h under either normoxic or hypoxic conditions. Equal protein amounts were introduced to SDS-PAGE, blotted, and incubated with either specific primary VEGF, HIF-1α, or β-actin antibody. β-actin was used as a loading control.

VEGFR-2 is a key regulator of VEGF-induced endothelial function. Therefore, the inhibitory effect of sMEK1 on VEGF-induced VEGFR-2 phosphorylation was evaluated *in vitro* in HUVECs. As revealed in Figure 2A, ectopic expression of sMEK1 inhibited VEGF-induced VEGFR-2 phosphorylation (Tyr-951) in a dose-dependent manner. In contrast, VEGFR-2 phosphorylation (Tyr-1175) and sMEK1-siRNA had no effect (Figure 2A and 2B). These data suggest that sMEK1 significantly decreased VEGFR-2 phosphorylation *in vitro* in HUVECs. We then assessed whether sMEK1 decreased p-VEGFR-2 levels via suppression of its kinase activity by investigating the effects of sMEK1 on VEGF-induced p-VEGFR-2 using ELISAs. The data confirmed that sMEK1 could inhibit VEGFR-2 kinase activity in a dose-dependent manner (Figure 2C). We next addressed whether sMEK1 controls VEGFR-2 transcriptional activity using a luciferase reporter-gene assay system and a construct containing the VEGFR-2 promoter fused to the luciferase gene. Luciferase activity was decreased by transient transfection of sMEK1 in a concentration-dependent manner (Figure 2D). Importantly, this observation was similarly able to reduce transcriptional activity in cancer cells such as SKOV-3 and MCF-7 (Supplementary Figure 1). These data confirm that sMEK1 plays an important role in regulating VEGFR-2 activity.

The expression of both VEGF and HIF-1α are regulated via PI3K/Akt signaling. In rapidly growing tumor, hypoxic conditions strongly activate the expression of the transcription factor HIF-1α, which in turn stimulates the expression of VEGF proteins in tumor cells. VEGF expression levels control the effects of other angiogenic regulators and therefore play major roles in the regulation of tumor angiogenesis. In order to disrupt new blood vessel formation in tumor, it is a key to inhibit the expression of the VEGF and HIF-1α proteins in tumor cells. To investigate whether sMEK1 inhibits the expression of HIF-1α under hypoxic conditions, we determined the time course of hypoxia-induced HIF-1α expression in sMEK1-transfected HUVECs. As shown in Figure 2E, HUVECs expressed little or no detectable HIF-1α protein under normoxic conditions (left panel). HIF-1α expression was activated rapidly when cells were seeded under hypoxic conditions, with maximal induction observed at 8∼16 h. HIF-1α levels remained consistent at 16 h, with a small reduction observed at 24 h (right panel). Subsequently, we measured the levels of HIF-1α and VEGF protein expression in HUVECs and SKOV-3 cancer cells (data not shown) exposed to normoxia or 1% O_2_ hypoxia and sMEK1 transfection. After a 16 h treatment, levels of HIF-1α and VEGF protein expression indicated that they were fully activated. In contrast, HIF-1α and VEGF expression levels were rapidly reduced after sMEK1 transfection (Figure 2F). Therefore, it may be suggested that hypoxia significantly promoted the levels of HIF-1α and VEGF expression, whereas sMEK1 suppressed their activation.

### sMEK1 inhibits VEGF-induced cell proliferation, migration, and tube formation

**Figure 3:**
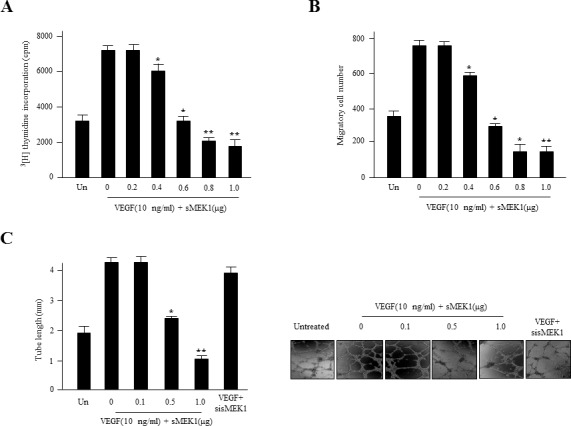
sMEK1 inhibits angiogenesis by abrogating endothelial cell proliferation, migration, and tube formation **A.** The growth inhibitory effects of sMEK1 on endothelial cell proliferation. HUVECs were seeded and incubated for 3 days with or without VEGF. The c.p.m. of [^3^H]thymidine was measured using a liquid scintillation counter. Each data point represents triplicate samples, and the bars indicate the means±SDs. *, *P* < 0.05; **, *P* < 0.01 *vs*. control. The experiments were repeated three times, with similar results. **B.** Boyden chamber-based migration assays were performed to address whether transfected sMEK1 modulates the effects of VEGF on endothelial cell migration. HUVECs were seeded into the top chamber of the Boyden Transwell chamber (pore size, 8 μm). HUVECs were fixed and then stained with H&E. The number of migrated cells was counted under a light microscope. Three independent experiments were conducted in triplicate. *, *P* < 0.05; **, *P* < 0.01 *vs*. control. **C.** HUVECs were transfected with sMEK1 and then grown on growth factor-reduced Matrigel in the presence or absence of 10 ng/ml VEGF. The formation of a tubular-like structured network was observed under an inverted microscope. The lengths of tubes were quantified and expressed as means±SDs. *, *P* < 0.05; **, *P* < 0.01 *vs*. control. Experiments were repeated three times, and a representative experiment is shown.

Angiogenesis is regulated by a number of pro- and anti-angiogenic components. For example, VEGF plays a pivotal role in tumor angiogenesis. To address whether sMEK1 has angiostatic effects, we assessed its effect on VEGF-stimulated endothelial cell proliferation. HUVECs were transfected with sMEK1, after which cell proliferation was measured using a [^3^H] thymidine incorporation assay. VEGF-stimulated DNA synthesis was compared between non-transfected cells and those transfected with control vector or sMEK1. Transfection with 0.8 μg sMEK1 decreased cell proliferation to ∼65% of that of the control transfected cells (Figure 3A). To investigate the potential roles of activated levels of sMEK1 in suppressing proliferation and VEGF-stimulated angiogenesis, we assessed HUVEC migration using a Transwell migration assay system. As expected, VEGF activated the migration of non-transfected and empty vector control-transfected cells compared with un-stimulated cells. However, the ectopic expression of sMEK1 inhibited VEGF-induced HUVEC migration (Figure 3B). Therefore, ectopically expressed sMEK1 inhibits several aspects of VEGF-induced angiogenesis *in vitro*, including endothelial cell proliferation and migration. In the latter process, endothelial cell capillary-like tubular structures are an important event to become elongated into tubes to form new blood vessels for angio­genesis. We then investigated the anti-angiogenic activities of sMEK1 on VEGF-induced tube formation using an *in vitro* angiogenesis model system. In the presence of sMEK1-siRNA, endothelial cells were almost none affected. In the presence of sMEK1, the linear structures of the capillary networks were disrupted (Figure 3C). Taken together, these findings suggest that sMEK1 controls VEGF-induced angiogenesis, including cell proliferation, migration, and tube formation, specifically.

### sMEK1 suppresses VEGF and HIF-1α expression in ovarian tumor cells

**Figure 4:**
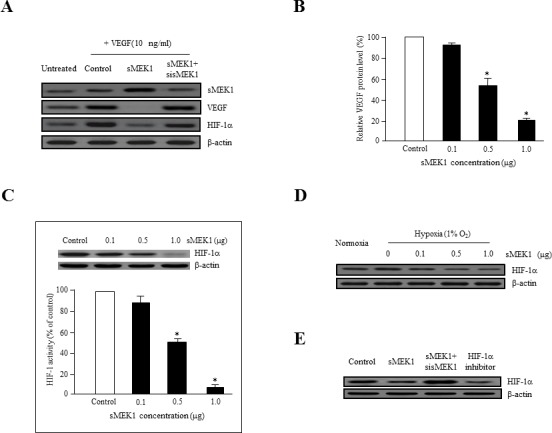
sMEK1 decreases VEGF and HIF-1α expression in ovarian carcinoma cells **A.** SKOV-3 ovarian cancer cells were incubated with 10 ng/ml VEGF activator and then transfected with the control (expression vector only), sMEK1, or sMEK1 plus sMEK1-siRNA. VEGF and HIF-1α expression was then visualized by immunoblot analysis. Three independent experiments revealed similar results. **B.** SKOV-3 cells were seeded and then transfected with increasing concentrations of sMEK1 (0∼1.0 μg) once they reached 80% confluence (exponentially growth). Protein concentrations were calculated using densitometric analysis and normalized to the levels of the loading control. The results shown are representative of at least three independent experiments. *, *P* < 0.05 *vs*. control. **C.** SKOV-3 cells were transfected with various concentrations of sMEK1, and nuclear extracts were harvested. HIF-1α activity was then measured using an ELISA kit (R&D Systems, Abingdon, UK). Three independent experiments were performed in triplicate. *, *P* < 0.05 *vs*. control. For Western blot analysis, we are used whole cell lysates. **D.** SKOV-3 cells were incubated for 16 h under hypoxic conditions (1% O_2_), and immunoblots were used to assess expression of HIF-1α. **E.** SKOV-3 cells were transfected with the control (expression vector only), sMEK1, sMEK1 plus sMEK1-siRNA, and Chetomin. After 24 h, the cells were harvested, lysed in ice-cold RIPA buffer, and analyzed by immunoblotting. Data are representative of three independent experiments.

To assess whether sMEK1 inhibits the expression of VEGF and HIF-1α via this signaling pathway, we analyzed VEGF expression in sMEK1-transfected SKOV-3 ovarian tumor cells. The ectopic expression of sMEK1 decreased VEGF expression significantly, whereas sMEK1-siRNA had no effect (Figure 4A). We also investigated the effect of sMEK1 on expression of HIF-1α, the major activator of VEGF expression. As shown in Figure 4A, sMEK1 notably decreased the expression of HIF-1α. In contrast, the effects of sMEK1 were completely prevented by transfection with sMEK1-siRNA. The expression of VEGF and HIF-1α was inhibited by sMEK1 in a concentration-dependent manner (Figure 4B and 4C). Next, the inhibitory effects of sMEK1 on HIF-1α expression under hypoxic conditions in SKOV-3 cancer cells were assessed. As shown in Figure 4D, sMEK1 decreased HIF-1α expression gradually in response to 1% O_2_ in a dose-dependent manner. In addition, VEGF-induced HIF-1α expression was inhibited by sMEK1 (Figure 4A and 4C). These results were similar to those exerted by Chetomin, a well-known HIF-1α inhibitor. Chetomin showed ∼10% lower activity than sMEK1 (Figure 4E). Collectively, these results suggest that hypoxia-induced VEGF protein levels were decreased significantly by sMEK1.

### sMEK1 suppresses PI3K, Akt, and eNOS phosphorylation

**Figure 5:**
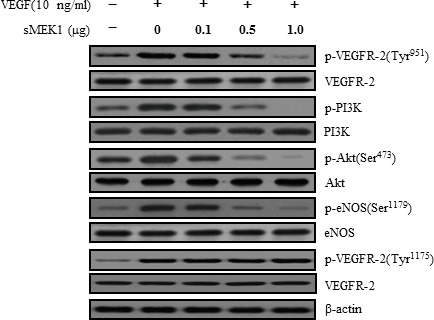
sMEK1 suppressed the phosphorylated proteins of the VEGFR-2/PI3K/eNOS signaling cascade Cell lysates were prepared from transfected SKOV-3 cancer cells or HUVECs (data not shown) and subjected to immunoblotting using primary antibodies specific to the phosphorylated or unphosphorylated forms of negative and positive regulators of Akt, including PI3K and eNOS. All experiments were repeated at least three times with similar results.

The direct physical interaction between VEGF and VEGFR-2 is important for activating the essential downstream components responsible for endothelial cell proliferation, migration, invasion, and survival, as well as tumor angiogenesis [50–52]. To further explore the molecular mechanism underlying the anti-angiogenic effects of sMEK1, we assessed the effect of sMEK1 on phosphorylation of the downstream modulators of the VEGFR-2/PI3K signaling pathway that regulate endothelial cell function. sMEK1 inhibited VEGF-stimulated phosphorylation of the VEGFR-2/PI3K signaling cascade, including Akt and endothelial nitric oxide synthetase (eNOS), but not VEGFR-2 (Tyr-1175) (Figure 5). Specially, we also confirmed whether sMEK1 affects VEGF-induced activation of Akt and eNOS complex. As presented in Supplementary Figure 2,sMEK1 inhibits VEGF-stimulated phosphorylation of Akt (Ser473 and Thr308). Generally, the activation of eNOS and endothelial tube network formation are dependent on Tyr-951 of VEGFR-2 and VEGFR-2 uses phospholipase Cγ1 (PLC-γ1) to stimulate these activities. However, VEGF receptor pathways converge on PI3K/Akt, which appears to be the common mediator of VEGF-induced eNOS activation and *in vitro* angiogenesis. VEGFR-2 activates eNOS through PLC-γ1. To explore whether protein abundance of VEGFR-2 was important in VEGF-induced VEGFR-2, cells were pre-treated with/without VEGF, transfected with sMEK1 or VEGFR-2 siRNA, respectively. Knockdown of VEGFR-2 recovered VEGF-induced downstream regulator expression as well as PI3K, phospholipase Cγ1 (PLC-γ1), and eNOS expression reduced by sMEK1 (Supplementary Figure 4 and 5). Therefore, sMEK1 might target the VEGFR-2 (Tyr-951)/PI3K/eNOS signaling pathway in ovarian tumorigenesis, subsequently inhibiting tumor angiogenesis and metastasis.

### sMEK1 inhibits tumor growth and angiogenesis in SKOV-3 xenografts in nude mice

**Figure 6:**
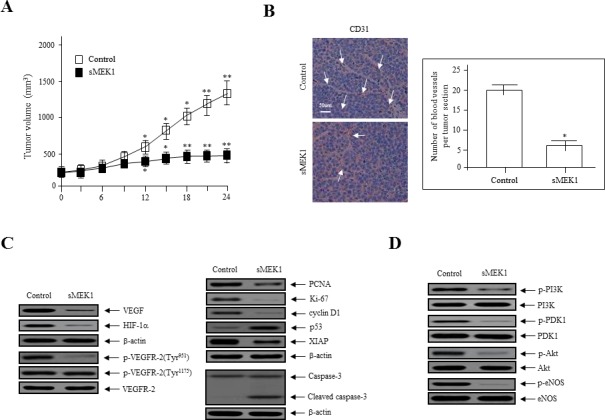
sMEK1 suppressed tumor growth by inhibiting angiogenesis *in vivo* **A.** Growth curves of human ovarian xenografts treated with sMEK1. Human SKOV-3 ovarian cancer cells were injected subcutaneously into nude mice and allowed to form tumors 70∼100 mm^3^ in size. Mice were injected i.p. with the control or sMEK1, and tumor size was calculated every 3 days. *, *P* < 0.05; **, *P* < 0.01 *vs*. control. **B.** Endothelial cells within paraffin-embedded tumor sections were stained using anti-CD31 antibodies. sMEK1-treated tumors exhibited an ∼3-fold reduction in the number of blood vessels stained with CD31. Bar = 50 μm. *, *P* < 0.05 *vs*. control. **C.** Soluble protein extracts were isolated from the tumors of xenografted mice and assayed by immunoblotting using antibodies against VEGF, HIF-1α, p-VEGFR-2, PCNA, Ki-67, cyclin D1, p53, XIAP, and caspase-3. VEGFR-2 and β-actin were used to verify equal loading amounts among the samples. **D.** sMEK1 treatment decreased the phosphorylation of major regulators of the PI3K/Akt signaling pathway. The levels of phospho-PI3K, phospho-PDK1, phospho-Akt, and phospho-eNOS in total tumor lysates were detected using immunoblot analysis. The unphosphorylated proteins were used as controls to verify equal loading amounts (PI3K, PDK1, Akt, and eNOS). The intensities of the protein bands were analyzed using densitometry.

To explore the direct anti-tumor and anti-angiogenic activities of sMEK1, we assessed its effects on tumor growth in ovarian cancer xenografts *in vivo*. SKOV-3 ovarian cancer cells were injected s.c. into nude mice to induce development of ovarian tumor xenografts. The tumor volume of the control group was actively enhanced after 14 days (mean volume, 100 mm^3^; Figure 6A). However, sMEK1-treated tumors were 65% smaller than those of the control group. Next, tumor tissue sections were stained with H&E, and the histological differences were analyzed. Treatment with sMEK1 caused no overt toxic effects in various organ tissue sections, and no toxic lesions were caused by sMEK1 (data not shown).

To confirm the anti-angiogenic effects of sMEK1 on tumor angiogenesis *in vivo*, we next counted the number of blood vessels in the vasculature using CD31 (PECAM-1) immunostaining of endothelial cells. A three-fold reduction in the number of CD31-stained blood vessels was observed in tumor sections from sMEK1-treated mice (Figure 6B). We then examined the expression of angiogenic factors. Immunoblotting demonstrated that VEGF and HIF-1α levels and VEGFR-2 phosphorylation were suppressed dramatically in sMEK1-treated tumors compared with the control. We also investigated the underlying biological mechanisms by which sMEK1 controls tumor growth. The expression of proliferating cell nuclear antigen (PCNA) and Ki-67 in the sMEK1-treated group was significantly lower than that in the control group. To assess whether sMEK1 induces apoptotic cell death, we next determined the proteolytic activity of caspase-3, which is the precursor of a major protease regulating apoptosis, via immunoblot analysis. The expression of cell death-associated proteins such as caspase-3 was increased significantly in sMEK1-treated tumors compared with the control (Figure 6C). These results suggest that sMEK1 could decrease tumor growth by inducing apoptotic cell death *in vivo*. In contrast, sMEK1 treatment did not induce cytotoxicity in HUVECs (Supplementary Figure 3). Finally, we used immunoblot analysis to assess the downstream targets of PI3K in sMEK1-treated tumors. sMEK1 treatment decreased the phosphorylation of PDK1, Akt, and eNOS compared with their respective total protein levels (Figure 6D). Collectively, these results suggest that sMEK1 potently inhibits angiogenesis and tumor growth *in vivo*.

## DISCUSSION

The current study demonstrated a mechanism whereby sMEK1 controls tumor angiogenesis by inhibiting p-eNOS (Ser-1179) and targeting the VEGFR-2/PI3K/eNOS signaling cascade in endothelial cells. VEGF phosphorylates VEGFR-2, and its downstream signaling activates endothelial nitric oxide synthetase (eNOS), which is a key modulator in the regulation of endothelial cell proliferation, migration, and survival [53]. Knockdown of VEGFR-2 recovered VEGF-induced downstream regulator expression as well as PI3K, PLC-γ1 and eNOS expression reduced by sMEK1 (Supplementary Figure 4). Generally, VEGF expression is regulated at the transcriptional level by HIF-1α under hypoxia- and growth factor-stimulated conditions [54, 55]. Many growth factors and hormones can promote PI3K/Akt-dependent phosphorylation of eNOS, which activates NOS and stimulates the subsequent increase in NO production [56, 57]. HIF-1α is regulated during several cellular biological responses and plays a pivotal role in angiogenesis and tumor progression [58]. After a 16 h treatment in HUVECs, levels of HIF-1α and VEGF protein expression indicated that they were fully activated. In contrast, HIF-1α and VEGF expression levels were rapidly reduced after sMEK1 transfection (Figure 2F). Therefore, it may be suggested that hypoxia significantly promoted the levels of HIF-1α and VEGF expression, whereas sMEK1 suppressed their activation. In addition, as presented in Figure 4A and 4C, VEGF-induced HIF-1α expression was remarkably inhibited by sMEK1. These effects were comparable to and ∼10% more effective than the known HIF-1α inhibitor Chetomin (Figure 4E). Furthermore, sMEK1 inhibited VEGF-induced phosphorylation Akt and eNOS, but not VEGFR-2 (Tyr-1175) (Figure 5). Therefore, sMEK1 targets the VEGFR-2 (Tyr-951)/PI3K/eNOS signaling pathway in ovarian tumorigenesis to inhibit angiogenesis and metastasis.

Increased expression of VEGF in ovarian cancers is associated with poor survival *in vivo*. In addition, VEGFR expression in ovarian cancer correlates with tumor grade and progression [59–62]. Adham et al. [63] reported a significantly elevated NRP-1:VEGFR-2 ratio and increased tumor grade in 80 cases of human epithelial ovarian cancer (EOC). In addition, VEGF receptors might control the biological function of fibroblasts in the tumor stroma [64]. Activated HIF-1α increases the proliferation, migration, and invasion of endothelial cells, as well as the formation of a capillary-like tubular structure network in aggressive solid tumors. Calvani et al. [65] reported that HIF-1α is a major mediator of the response of endothelial cells to hypoxia, confirming its potential role as a therapeutic target in carcinoma cells. The HIF-1α downstream target VEGF also plays an important role in tumor angiogenesis and is an attractive chemotherapeutic target [66].

In conclusion, this study revealed that sMEK1 decreased HIF-1α protein expression by inhibiting VEGF-induced VEGFR-2, PI3K, Akt, and eNOS phosphorylation. eNOS plays a central role in maintaining vascular integrity [67] and is activated by VEGF and shears stress via the VEGFR-2 [68, 69]. It is also known that nitric oxide (NO) is a key regulator of endothelial cell migration and VEGF-induced angiogenesis [70]. Here, sMEK1 exerted anti-angiogenic effects by decreasing HIF-1α and, consequently, *VEGF* expression. Therefore, this study demonstrated that sMEK1 exerts potential anti-angiogenic effects by inhibiting VEGF-induced HIF-1α and VEGF protein expression in tumors. In addition, we propose a schematic diagram in Supplementary Figure 5 (right panel) that sMEK1 tumor suppressor protein interrupts the phosphorylation of signaling components such as HIF-1α, p-eNOS, p-VEGFR-2 and p-Akt. It can be concluded sMEK1 can suppress tumor growth through the inhibition of tumor angiogenesis in the cellular biological/physiological condition through the targeting of the VEGFR-2/Akt/eNOS signaling pathway.

## MATERIALS AND METHODS

### Culture conditions, animals and antibodies

Human SKOV-3 ovarian carcinoma cells were purchased from the American Type Culture Collection (ATCC, Manassas, VA) and were maintained in DMEM media (Life Technologies, Gaithersburg, MD) supplemented with either 10% heat-inactivated fetal bovine serum (FBS), including penicillin (100 U/ml) and streptomycin (100 μg/ml). HUVECs (human umbilical vein endothelial cells) were obtained from Clonetics (Walkersville, MD), and were grown on 0.3% gelatin (Sigma, St. Louis, MO) coated dishes using the EGM-2 BulletKit medium (Clonetics). Cells were seeded in a humidified atmosphere containing 5% CO_2_ at 37°C. Specific pathogen-free BALB/c-nu/nu mice (5∼6 week old) were supplied by Orientbio (Sungnam, Korea). All animal studies were approved by the Institutional Animal Care and Use Committee (IACUC) at the Research Institute of the National Cancer Center. The primary antibodies used in this study were anti-sMEK1 (Abcam, Cambridge, UK), anti-VEGFR-1, anti-VEGFR-2, anti-phospho-VEGFR-2(Tyr-951), anti-phospho-VEGFR-2(Tyr-1175), anti-eNOS, anti-phospho-eNOS(Ser-1179), anti-p53, anti-caspase-3 (Cell Signaling, Beverly, MA), anti-VEGF, anti-CD31 (PECAM-1), anti-Ki67 (Ab-1; Oncogene, Cambridge, MA), anti-XIAP (BD Biosciences, San Jose, CA), anti-HIF-1α, anti-PI3K, anti-phospho-PI3K, anti-PLC-γ1, anti-phospho-PLC-γ1(Tyr-783), anti-phospho-PDK1, anti-PDK1, anti-Akt, anti-phospho-Akt (Ser-473 and Thr-308), anti-GSK-3β, anti-phospho-GSK-3β(Ser-9), anti-cyclin D1 (Santa Cruz Biotechnology, Santa Cruz, CA), anti-PCNA (Dako, Denmark), and β-actin (Sigma).

### Co-immunoprecipitation

cDNA encoding human sMEK1 was introduced into pcDNA3.1/Flag expression vector (Invitrogen) and digested with *Eco*RI and *Xho*I (pcDNA3.1/Flag-sMEK1). The human VEGFR-1 and VEGFR-2 cDNA was ligated into pcDNA3.1 expression vector (Invitrogen) using *Eco*RI and *Xho*I (pcDNA3.1-VEGFR-1 and pcDNA3.1-VEGFR-2). For co-immunoprecipitation, HEK293T cells were co-transfected with cDNA constructs of pcDNA3.1/Flag-sMEK1 and pcDNA3.1-VEGFR-1 or pcDNA3.1-VEGFR-2 using Lipofectamine 2000 transfection reagent (Invitrogen). Cells were trypsinized and then centrifuged. Cell pellets were washed in PBS, resuspended in lysis buffer (50 mM Tris/HCl, pH 7.2, 150 mM NaCl, 1% Triton X-100, protease inhibitor cocktail containing 1 μg/ml leupeptin, 1 μg/ml pepstatin, 2 μg/ml aprotinin, 200 μg/ml PMSF). Lysates were then incubated with anti-Flag antibody (Santa Cruz) and precipitated with protein A-agarose (Amersham). The precipitated proteins were separated by SDS-PAGE electrophoresis, transferred onto Immobilon P membrane (Millipore corporation, Billerica, MA), and immunoblotted with anti-sMEK1, anti-VEGFR-1, or anti-VEGFR-2 antibody using the ECL system (Amersham).

### Subcloning of truncated mutants of VEGFR-2

Three truncated fragments (Met^1^-Gly^800^, Leu^801^-Leu^1000^, Thr^1001^-Val^1356^) of VEGFR-2 were isolated by polymerase chain reaction (PCR) for *in vivo* assay. PCR products spanning each fragment were cloned into the *Eco*RI and *Xho*I sites of the pJG4-5. The cDNAs encoding pJG4-5 fusion proteins were introduced into the competent yeast cells that already contained pGilda-sMEK1 and the transformants were selected for the tryptophan prototrophy (plasmid marker) on synthetic medium (Ura^−^, His^−^, Trp^−^) containing 2% (w/v) glucose. The activity of the interaction between sMEK1 and VEGFR-2 was confirmed by measuring the relative expression level of β-galactosidase. The β-galactosidase assay was measured according to the previously described method [71].

### *In vitro* VEGFR-2 kinase assay

*In vitro* VEGFR-2 tyrosine kinase activity was evaluated using the HTScan VEGFR-2 kinase assay kit (Cell Signaling) together with colorimetric ELISA, as described previously [72, 73]. The final reaction consisted of 60 mmol/L HEPES (pH 7.5), 5 mmol/L MgCl_2_, 5 mmol/L MnCl_2_, 3 μmol/L Na_3_VO_4_, 1.25 mmol/L DTT, 20 μmol/L ATP, 1.5 μmol/L substrate peptide, 100 ng VEGFR kinase, and sMEK1 at the indicated concentrations. The results are expressed as the percentages of kinase activity relative to the control (100%), and the IC_50_ value was defined as the sMEK1 plasmid concentration resulting in 50% suppression of enzyme activity.

### Luciferase reporter-gene assay

To measure the VEGFR-2 promoter activity, a VEGFR-2 promoter fragment was subcloned from human placental cDNA by PCR using primers (forward primer: 5′-TAGCGAGCTCTGCCACAAGAAGTCCACACA-3′, reverse primer: 5′-CACCCGACCTGTCTGCCTTCC-3′). The domain involving the VEGFR-2 promoter (from −887 to +295) region was eluted from the pCR2.1-TOPO (Invitrogen) cloning vector with *Sac*I and *Xho*I restriction enzyme sites and was then introduced into the same cloning sites of the pGL3 luciferase reporter expression vector (Promega, Madison, WI). The resulting fragments were separated by 1.2% agarose gel electrophoresis and were performed to confirm correct insertion orientation using automatic sequencing (ABI 373, PerkinElmer Life Sciences). *In vitro* VEGFR-2 promoter activity was performed as previously reported [74]. In brief, cells at 80% confluency were transiently transfected with each indicated reporter plasmid. After lysis, lysates were cleared with centrifugation for 20 min at 12,000 rpm and cell extracts were incubated with the luciferase substrate reagent at room temperature for 30 min. Then, a 5 μl aliquot of each sample was placed into the MicroLumat Plus LB96V luminometer.

### Cell migration assay

Cell migration assay was performed using 8-μm pore-size Transwells (Corning Costar, Cambridge, MA) as described previously [74]. Briefly, HUVECs suspended in DMEM containing 0.1% BSA (Sigma) were seeded onto Matrigel (BD Biosciences, San Jose, CA) in 24-well plates as a monolayer at 4.0×10^4^ cells per well. For the migration assays, the lower surface of each filter was coated with 10 μg of gelatin. M199 containing 1% FBS and 25 ng/ml VEGF was placed in the lower chamber, and the cells were allowed to migrate in a 5% CO_2_ incubator at 37°C for 24 h. After incubation, the cells were fixed and stained with hematoxylin and eosin (Sigma) according to the manufacturer's protocol. Non-migrant cells in the upper chamber were removed by wiping with a cotton swab. Cells that migrated to the lower side of the filter were counted under an inverted microscope, and the mean value of eight fields was measured.

### Endothelial cell tubular structure formation assay

Growth Factor Reduced Matrigel (200 μl of 10 mg/ml; BD Biosciences) was added to each well of a 24-well plate and incubated for 30 min at 37°C to allow gel formation. The seeded cells were then incubated with or without 10 ng/ml VEGF for 48 h in M199 containing 1% FBS. Morphological changes were photographed at 40× magnification. Capillary-like tubular structures were observed using an inverted microscope equipped with a digital CCD camera (Zeiss) and quantified using ImageLab imaging software (MCM Design, Copenhagen, Denmark).

### Hypoxic experimental conditions

HUVECs were seeded at a density of 2.5×10^5^ /well in 24-well plates and then exposed to hypoxic (1% O_2_) or normoxic (21% O_2_) conditions for 0, 4, 8, 16, or 24 h. Cells were harvested and lysed in lysis buffer supplemented with protease inhibitors. Supernatants were collected after centrifugation at 4,000 × *g* for 10 min to remove cellular debris. The protein concentrations in the samples were then assessed using a Bradford protein assay kit (Bio-Rad, Hercules, CA). Equal amounts of supernatant were loaded onto gels to assess the expression of the soluble form of sMEK1 via immunoblotting.

### Xenograft mouse model and immunohistochemistry

Briefly, human SKOV-3 ovarian carcinoma cells (2.1×10^6^) were injected subcutaneously (s.c.) into 5∼6-week-old BALB/c-nu/nu mice and allowed to form tumors 70∼100 mm^3^ in size. Mice were treated by intraperitoneal (i.p.) injection with control or sMEK1. Tumor volume was measured in three dimensions using calipers and calculated by the following formula: tumor volume (mm^3^) = (*a* × *b*^2^)/2, where *a* = length in mm and *b* = width in mm. Body weight was measured every other day. Tumors were then excised and weighed, and 1/2 were frozen and 1/2 were fixed in 10% neutral-buffered formalin (NBF) to generate paraffin blocks for sectioning, hematoxylin and eosin (H&E) staining, and immunohistochemistry.

For immunohistochemistry, tumor samples from xenografted mice were harvested, fixed in 10% NBF (Sigma), and serially sectioned (5-μm thickness). Slides were then deparaffinized and rehydrated in xylene and a graded alcohol series, respectively, and rinsed with phosphate buffer saline (PBS). The slides were incubated in 5% hydrogen peroxide in methanol for 20 min to block endogenous peroxidase activity. They were then incubated with a saturating concentration of anti-mouse CD31 (platelet-derived endothelial cell adhesion molecule; PECAM-1) (Abcam) antibody overnight at 4°C, followed by a streptavidin-peroxidase complex at room temperature for 1 h. Microvessel density was quantified in five randomly selected individual tumor fields (at ×40 magnification) from each sample. The number of microvessels was also counted under a high-power microscope (at ×400 magnification). Additional slides were stained with H&E (Sigma) according to the manufacturer's instructions. All immunochemical analyses were performed using an Axiophot 2 apparatus (Carl Zeiss MicroImaging Inc., Thornwood, NY).

For immunoblot anlysis, protein lysates were prepared from homogenized frozen tumor tissues, and a Bradford protein assay was used to determine protein concentration. Equal amounts of protein (20 μg) were loaded onto 8∼12% SDS-PAGE.

### Statistical analysis

The data represented is the means±SDs and were analyzed statistically using Student's *t*-test for comparisons between two groups. Significant differences of 95% confidence (*P* < 0.05) are depicted with an asterisk (*) on each graph.

## SUPPLEMENTARY MATERIAL FIGURES


