# Supplementary material for: sMEK1 inhibits endothelial cell proliferation by attenuating VEGFR-2-dependent-Akt/eNOS/HIF-1α signaling pathways
Source: Oncotarget. 2015 Sep 10;6(31):31830–43. doi: 10.18632/oncotarget.5570 (PMC4741643; doi:10.18632/oncotarget.5570)
Supplement: Supplementary file 1 [file oncotarget-06-31830-s001.pdf]

sMEK1 inhibits endothelial cell proliferation by attenuating VEGFR-2-dependent-Akt/eNOS/HIF-1α signaling pathways

Supplementary Material

MATERIALS AND METHODS

Apoptosis assay

For the analysis of the DNA content by use of a flow cytometry (FACS), HUVECs were grown onto chamber slides at a density of 4.5×10^4 cells per well and were then transfected with sMEK1 for the indicated times relative to that of control cells (untransfected). Cells were incubated with FITC-labeled Annexin V and propidium iodide for 15 min according to the manufacturer's protocols (BD PharMingen, Mississauga, ON) and then were measured with a flow cytometer (FACScalibur, Becton Dickinson, Franklin Lakes, NJ).

RNA interference (RNAi)

Small interfering RNA (siRNA) oligonucleotide that target vascular endothelial growth factor receptor 2 (VEGFR-2) (ON-TARGET *plus* SMARTpool) and a non-targeting siRNA pool were obtained from Millipore (Billerica, MA) and resuspended according to the supplier's protocols. For transfection, cells were transfected with the oligonucleotides at a final concentration of 100 nM using Lipofectamine™ 2000 Reagent (Invitrogen, Carlsbad, CA) according to the manufacturer's instruction.

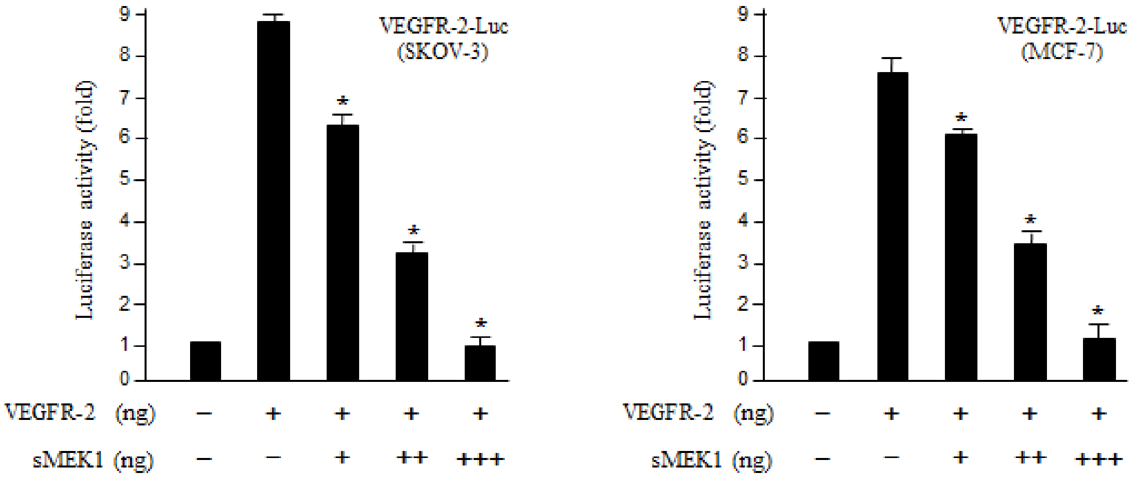

Fig S1: Inhibition of VEGFR-2-dependent transcription by sMEK1. Cancer cells (SKOV-3 and MCF-7) were co-transfected with 500 ng of VEGFR-2-Luc, 500 ng of a VEGFR-2 expression plasmid (pcDNA3.1/VEGFR-2), and increasing concentrations of sMEK1 (50, 250, and 500 ng). Each data point represents triplicate samples, and the bars indicate means±SDs. *, P<0.05 vs. control. The experiments were repeated three times with similar results.

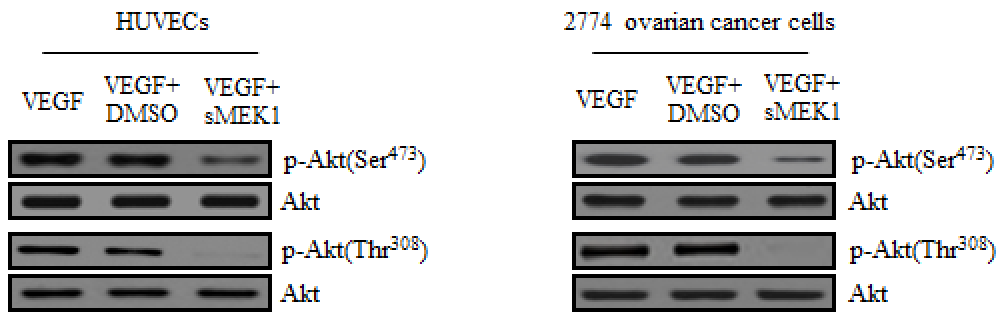

Fig S2: sMEK1 decreases VEGF-induced Akt phosphorylation. Cells (HUVECs and 2774 ovarian cancer) were treated with VEGF alone, VEGF plus DMSO, and VEGF plus sMEK1. Total cell extracts were assessed by immunoblot analysis with the indicated phosphor-specific or total-protein antibodies. Results shown are representative of three independent experiments.

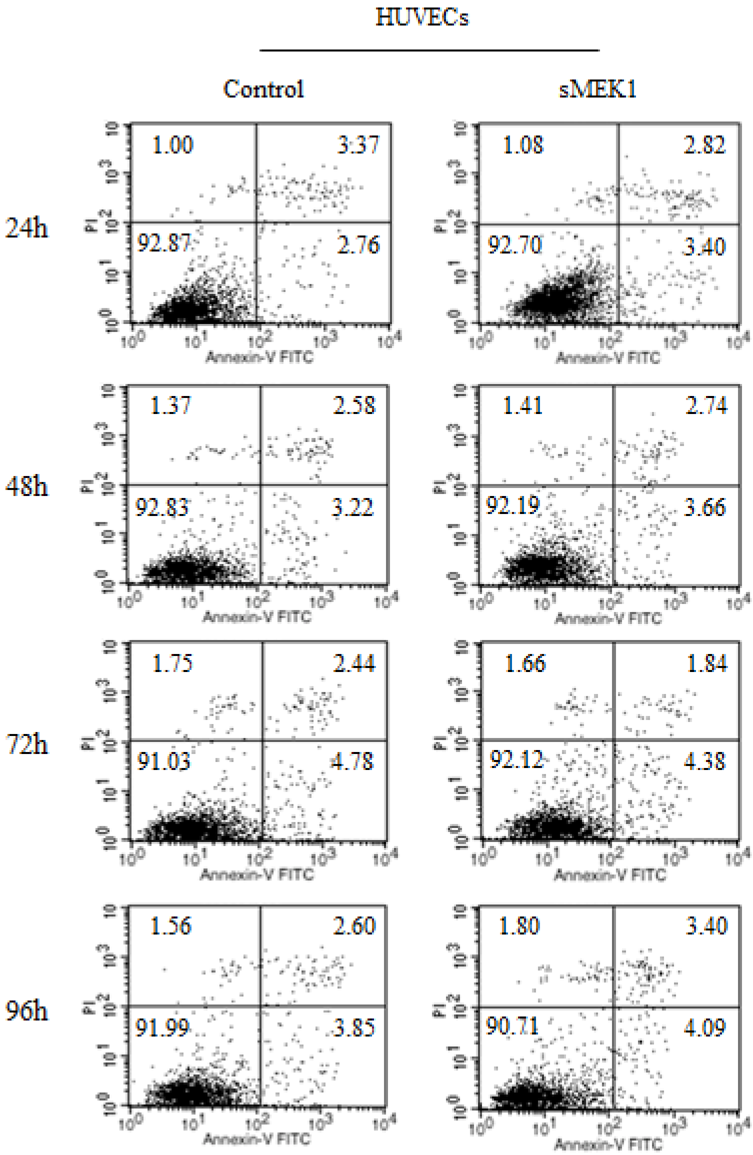

Fig S3: The growth-inhibitory effects of sMEK1 on HUVECs. HUVECs were transfected with increasing times of sMEK1, and then analyzed by fluorescein isothiocyanate (FITC)-labeled Annexin V assay. All data shown are representative of three independent experiments.

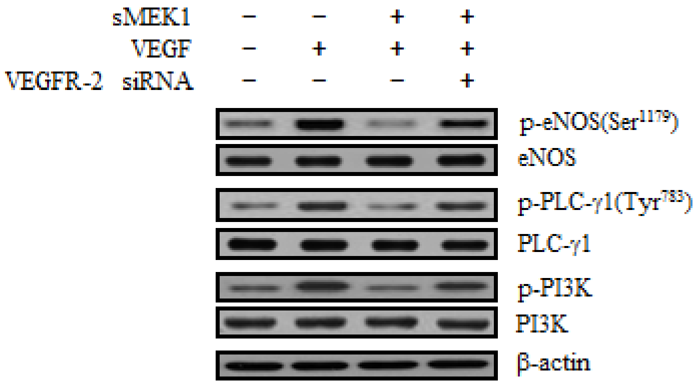

Fig S4: sMEK1 suppresses VEGF-stimulated PI3K- and downstream signaling components expression of VEGFR-2. SKOV-3 cells transiently transfected with VEGFR-2 siRNA were incubated with or without 10 ng/ml VEGF. Cells lysates were harvested and analyzed with the indicated antibodies by Western blot analysis. Knockdown of VEGFR-2 recovers VEGF-stimulated downstream regulators expression reduced by sMEK1. Three independent experiments were conducted in triplicate.

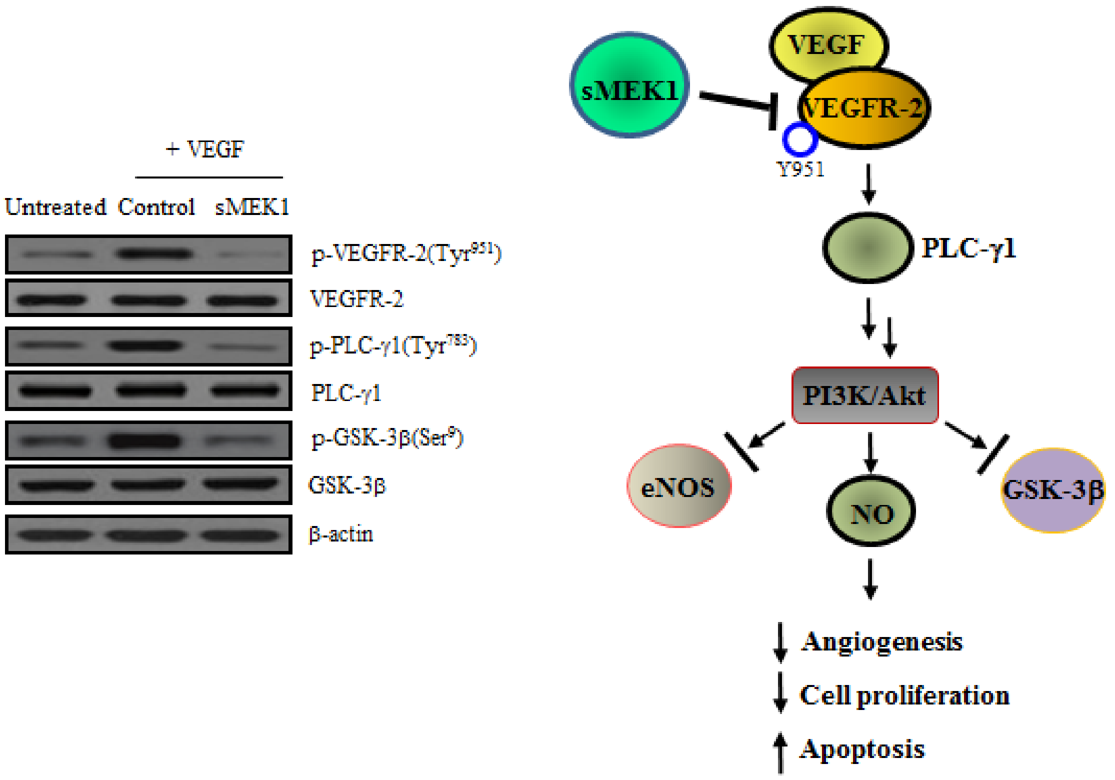

Fig S5: sMEK1 inhibits VEGF-induced VEGFR-2, PLC-γ1, and GSK-3β phosphorylation. Cells were treated with VEGF untreated, VEGF alone, or VEGF plus sMEK1. VEGF-induced phosphorylation of VEGFR-2, PLC-γ1, and GSK-3β was detected with anti-phospho-VEGFR-2(Tyr^{951}), anti-phospho-PLC-γ1(Tyr^{783}), and anti-phospho-GSK-3β(Ser^{9}) antibody, respectively. Unphosphorylated blot was used as loading control (indicated as VEGFR-2, PLC-γ1, GSK-3β, and β-actin). Results shown are representative of three independent experiments (left panel). Schematic diagrams for interruption of the phosphorylation of signaling components such as HIF-1α, p-eNOS, p-VEGFR-2 and p-Akt by the sMEK1 tumor suppressor protein (right panel).
